# Human insulin polymorphism upon ligand binding and pH variation: the case of 4-ethylresorcinol

**DOI:** 10.1107/S2052252515013159

**Published:** 2015-08-04

**Authors:** S. Fili, A. Valmas, M. Norrman, G. Schluckebier, D. Beckers, T. Degen, J. Wright, A. Fitch, F. Gozzo, A. E. Giannopoulou, F. Karavassili, I. Margiolaki

**Affiliations:** a Section of Genetics, Cell Biology and Development, Department of Biology, University of Patras, GR-26500 Patras, Greece; b Diabetes Protein Engineering, Novo Nordisk A/S, Novo Nordisk Park, DK-2760 Malov, Denmark; c PANalytical B.V., Lelyweg 1, 7602 EA Almelo, The Netherlands; d European Synchrotron Radiation Facility, CS40220, F-38043 Grenoble CEDEX 9, France; e Excelsus Structural Solutions, Belgium

**Keywords:** powder diffraction, human insulin, diabetes, synchrotron radiation, X-ray diffraction, pH variation, 4-ethylresorcinol

## Abstract

This study focuses on the effects of the organic ligand 4-ethylresorcinol on the crystal structure of human insulin using powder X-ray crystallography. For this purpose, systematic crystallization experiments have been conducted in the presence of the organic ligand and zinc ions within the pH range 4.50–8.20, while observing crystallization behaviour around the isoelectric point of insulin. High-throughput crystal screening was performed using a laboratory X-ray diffraction system. The most representative samples were selected for synchrotron X-ray diffraction measurements, which took place at the European Synchrotron Radiation Facility (ESRF) and the Swiss Light Source (SLS). Four different crystalline polymorphs have been identified. Among these, two new phases with monoclinic symmetry have been found, which are targets for the future development of microcrystalline insulin drugs.

## Abstract 2

This study focuses on the effects of the organic ligand 4-ethylresorcinol on the crystal symmetry and lattice dimensions of human insulin using powder X-ray crystallography.

## 1. Introduction   

Diabetes is rapidly reaching epidemic proportions, affecting 150 million people worldwide and being projected to double in prevalence by 2025 (Zimmet *et al.*, 2001 ▸; Carulli *et al.*, 2005 ▸; Mogensen & Zimmet, 2002 ▸; Emami-Riedmaier *et al.*, 2015 ▸). Since many cases go undiagnosed, these figures are likely to be an underestimate of its true prevalence. Left uncontrolled, diabetes can lead to coronary heart disease, kidney failure, blindness, limb amputations and premature death. The hallmark characteristic of type I diabetes is a lack of insulin.

Insulin consists of two distinct chains (A and B) which are linked together by two disulfide bonds. There is an additional intra-chain linkage in the A chain (Ryle *et al.*, 1955 ▸). All three disulfide bonds are essential for the receptor-binding activity of insulin (Chang *et al.*, 2003 ▸). Insulin in its monomeric form is an active hormone. However, the molecules tend to form dimers and in the presence of Zn^2+^ ions they form hexamers. The hexameric form is not active and acts as a storage form which provides the organism with the hormone when required. The addition of allosteric ligands (for example phenol and chloride ions) to insulin compositions is widely used to modify the pharmacodynamics and stability of pharma­ceutical preparations (DeFelippis *et al.*, 2001 ▸). Different formulations of the hormone are absorbed at different rates and have varying durations of action.

Crystallization has always been a key activity since the protein is often administered by the subcutaneous injection of crystalline formulations. Microcrystalline insulin solutions are already widely used in pharmaceutical formulations because of their stability and prolonged action. These pharmaceutical compositions usually contain insulin, zinc and a phenolic binder. Phenol results in further stability of the tertiary structure of the protein (Brange *et al.*, 1992 ▸). Microcrystalline drugs exhibit certain advantages over formulations in solution. Higher concentrations of the drug can be achieved in crystals compared with the amorphous form in solution. Additional advantages include low viscosity of the composition and controlled release of the protein as the crystals gradually dissolve in the body (Basu *et al.*, 2004 ▸). Crystallization of proteins is also less costly than lyophilization (Collings *et al.*, 2010 ▸). Of course, the crystal supernatant should only contain additives that are approved as nontoxic. When packaged in a crystalline form, proteins display greater stability and resistance to chemical changes and are less sensitive to denaturation of their three-dimensional structure. Finally, crystalline proteins are often protected against proteolytic enzymes (Halban *et al.*, 1987 ▸).

An important aspect that affects crystallinity is accurate control of the pH, as previous studies have reported that crystal morphology is often pH-dependent (McPherson, 1985 ▸; Farr *et al.*, 1998 ▸). Protein solubility reaches a minimum at the isoelectric point (pI) and increases at both lower and higher pH values. The probability of yielding high-quality protein crystals, in terms of morphology, has been suggested to increase around the pI owing to the minimum protein solubility. However, numerous studies have shown that this is not always the case (Farr *et al.*, 1998 ▸; Kantardjieff & Rupp, 2004 ▸). On the other hand, pH ranges, which are different for each protein, do exist where either lysine and arginine side chains begin to lose their positive charge or where, in an alternative case, the carboxyl groups of aspartic and glutamic acid side chains begin to lose their negative charge (McPherson, 1995 ▸). This partial neutralization of the molecule disrupts the formation of salt bridges between protein molecules and thus decreases the crystallization rate. A lower degree of nucleation is likely to result in fewer but larger and better-formed crystals owing to the control of rapid crystal growth at low and high pH.

Over the years, insulin has been crystallized and characterized in a number of crystal systems. Thanks to the three-dimensional insight obtained from dozens of crystal structures of the wild type (Hodgkin, 1971 ▸), mutants (Whittingham *et al.*, 1998 ▸) and complexes with zinc ions and small molecules such as phenol-based ligands (Derewenda *et al.*, 1989 ▸; Von Dreele *et al.*, 2000 ▸; Norrman, 2007 ▸; Karavassili *et al.*, 2012 ▸; Margiolaki *et al.*, 2013 ▸; Valmas *et al.*, 2015 ▸), it has been possible to fine-tune the kinetics of insulin dissociation. The resulting availability of a variety of insulin preparations with rapid or prolonged action profiles has improved the quality of life of millions of people (Brange, 1997 ▸). In the presence of Zn^2+^ ions, insulin crystallizes as a hexamer consisting of three dimers related by a crystallographic threefold axis (Bhatnagar *et al.*, 2006 ▸; Norrman, 2007 ▸). The pharmaceutical formulations used for treatment are typically mixtures of crystalline and amorphous protein, which are injected subcutaneously, resulting in long-term hormone action owing to the slow dissolution of the protein crystals (Norrman & Schluckebier, 2007 ▸). Thus, insulin has been crystallized under several conditions in order to determine how the morphology of the crystals affects its release into the bloodstream.

To date, several different polymorphs have been identified and most of them are found to belong to the monoclinic, rhombohedral, tetragonal and cubic symmetries. Besides this type of polymorphism, different conformations of the B chain have been found, which subsequently lead to different conformations of the hexamer. Hence, three different hexameric conformations occur, which are denoted T_6_, T_3_R_3_^f^ and R_6_ depending on the type of B chains that they contain (Bhatnagar *et al.*, 2006 ▸; Frankaer *et al.*, 2012 ▸). The B-chain conformation depends on the zinc and chloride ion content of the crystallization solution, as well as that of other ligands (Adams *et al.*, 1969 ▸; Bentley *et al.*, 1976 ▸; Derewenda *et al.*, 1989 ▸). Typically, in the absence of high chloride ion concentrations or phenolic derivatives the T_6_ hexamer is produced, while at high chloride or thiocyanate concentrations the T_3_R_3_^f^ hexamer is produced (Smith *et al.*, 2000 ▸). However, the addition of phenolic derivatives such as phenol or resorcinol will drive the transformation from the T to the R state, resulting in monoclinic crystals containing R_6_ hexamers (Derewenda *et al.*, 1989 ▸).

In this study, we investigate the effect of the resorcinol-based ligand 4-ethylresorcinol on the crystallization of human insulin (HI) as a function of pH. Resorcinol and its derivatives are extensively used as antiseptics and disinfectants in pharmaceutical formulations. The crystal polymorphism must be fully characterized in order to produce a drug in a crystalline form. In this case, X-ray powder diffraction (XRPD) is the most appropriate tool for the characterization of the various polymorphs, providing information on the microcrystalline samples (Margiolaki & Wright, 2008 ▸; Margiolaki, 2016 ▸; Karavassili & Margiolaki, 2015 ▸). Thereby, characterization of the insulin polymorphs produced based on this specific resorcinol ligand was performed.

## 2. Experimental   

### 2.1. Crystallization   

Purified recombinant HI was provided by Novo Nordisk and crystallization was performed using the salting-out method in batch. A stock protein solution was prepared by adding 401.2 mg as-received freeze-dried insulin to 21 ml double-distilled H_2_O along with 2.5 ml 0.01 *M* zinc acetate solution, resulting in a protein concentration of 17.07 mg ml^−1^. Two series of samples were prepared, in which we followed the same procedure, and each one was measured in a separate diffraction experiment. Each crystallization series was performed in a pH range of approximately 4.00–8.50, keeping the other parameters constant, in order to investigate the effect of pH on the crystal forms obtained. The crystallization series are denoted Series 1 and Series 2 throughout this article.

For preparation of the protein mixture, 17 ml of the protein solution was extracted and placed in a Falcon tube along with 0.510 ml 2 *M* 4-ethylresorcinol diluted in DMSO. Finally, after 5 min of incubation, 0.2 ml 1 *M* sodium thiocyanate was added to the protein mixture. Furthermore, we prepared stock buffers of 2 *M* Na_2_HPO_4_ and 2 *M* KH_2_PO_4_. These solutions were mixed in order to produce a pH buffer range in the region of interest between 4.5 and 8.7 with steps of roughly 0.5 units for Series 1 and ∼0.3 units for Series 2. Each sample was produced by mixing 1 ml protein mixture with 250 µl pH-buffer mixture in an Eppendorf tube, giving a final protein concentration of 13.11 mg ml^−1^. The final concentration of 4-ethylresorcinol in each sample was 46 m*M*.

**Figure 1:**
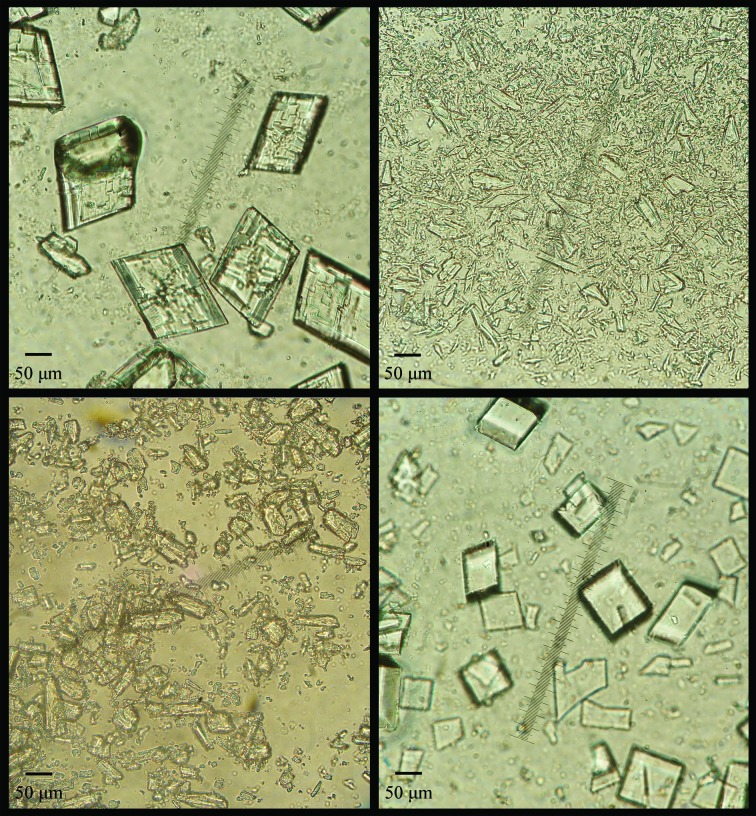
Polycrystalline samples of HI cocrystallized with 4-ethylresorcinol corresponding to pH 5.00 (upper left), 5.80 (upper right), 5.97 (lower left) and 7.37 (lower right). Each crystalline phase corresponds to a different symmetry, as shown by our analysis.

The samples were left to crystallize in an incubator at 298 K. After ∼48 h, polycrystalline precipitates appeared (Fig. 1 ▸). The pH of the crystallization solutions was measured before crystallization as well as after the diffraction experiments and a very small shift (∼0.2) towards higher pH levels was observed for the majority of the samples. The reported pH values in this paper correspond to the mean values of the above measurements.

### 2.2. Data collection and processing   

High-throughput crystal screening was performed *via* the collection of XRPD data in our laboratory using an X’Pert PRO diffractometer (PANalytical) at room temperature (RT) (λ = 1.541874 Å). During data collection employing the laboratory diffracto­meter, no significant radiation damage was observed after 24 h of measurements.

**Figure 2:**
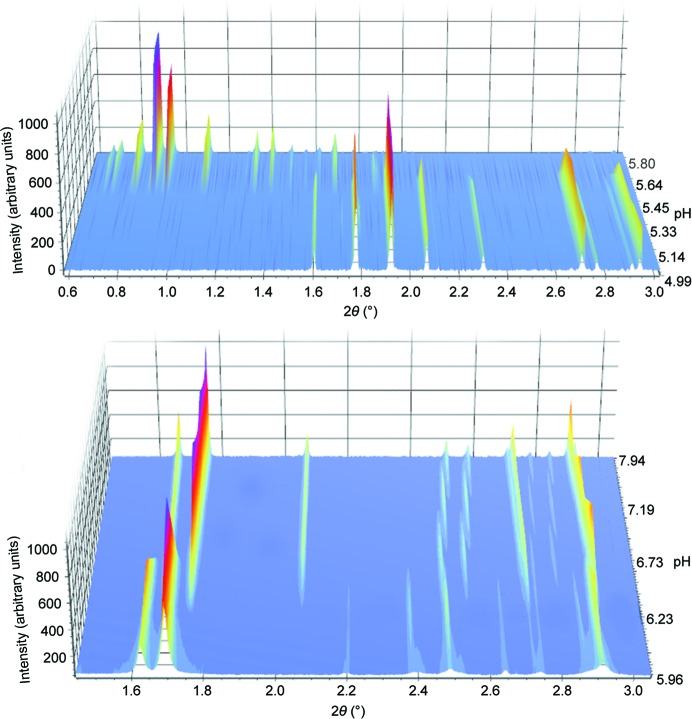
Top, data sets for HI cocrystallized with 4-ethylresorcinol corresponding to the *P*2_1(γ)_ (pH 4.99–5.45) and *P*2_1(α)_ (pH 5.64–5.80) polymorphs. Bottom, data sets for HI cocrystallized with 4-ethylresorcinol corresponding to the *C*2 (pH 5.96–6.23) and *P*2_1(β)_ (pH 6.73–7.94) polymorphs. Data were collected on ID31 at ESRF [λ = 1.29994 (3) Å, RT].

High-resolution XRPD data were collected (Fig. 2 ▸) on beamline ID31 at the European Synchrotron Radiation Facility (ESRF) in Grenoble (Fitch, 2004 ▸). After loading, the capillaries were centrifuged in order to enhance the crystal packing. The capillary tubes were also spun during the measurements to avoid preferred orientation effects. Each sample was measured at several positions in order to counterbalance the radiation damage caused by the intense synchrotron beam, and several scans were collected at each position. Thus, the samples were translated by 2 mm every 4 min, exposing a fresh region of protein powder. The first scans at each position were combined in order to improve the counting statistics. The subsequent scans were only used in order to follow the evolution of the unit-cell parameters under exposure to X-ray radiation.

**Figure 3:**
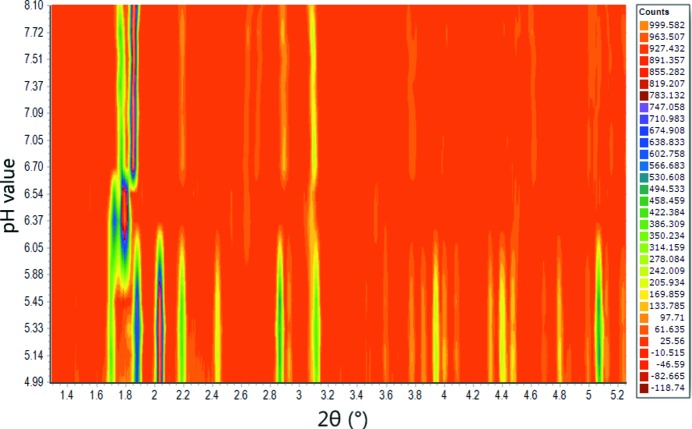
Colour representation of XRPD data from the pH-variation experiment (pH 4.99–8.10) for HI cocrystallized with 4-ethylresorcinol. Data were collected on MS-X04SA at SLS [λ = 1.37807 (15) Å, RT]. Phase transitions are observed at pH values of 5.88 and 6.70.

Additional measurements were performed on the materials science beamline MS-X04SA (Fig. 3 ▸) at the Swiss Light Source (SLS) in Villigen (Willmott *et al.*, 2013 ▸). The samples were measured at RT using a wavelength of 1.37807 (15) Å and a position-sensitive Mythen detector. Each sample was loaded into a borosilicate glass capillary tube of 80 mm in length and 0.5 mm in diameter. Each sample was measured at several positions, with each scan lasting 2 s. Many scans were also collected per position, giving a total exposure time of 70 s. All data-collection parameters are listed in Table 1 ▸.

The diffraction patterns were typically indexed using *DASH* (David *et al.*, 2006 ▸) employing the fitted positions of at least the first 20 reflections of the high angular resolution powder diffraction profiles. From the extracted data, we were able to determine the symmetry and unit-cell parameters for all samples. In order to obtain accurate values of the unit-cell parameters and characterize the peak shape and background coefficients without a structural model, Pawley fits (Pawley, 1981 ▸) were performed using *PRODD* (Wright, 2004 ▸).

#### 2.2.1. Cluster analysis   

Analysis of the large amounts of data from the high-throughput screening of protein–ligand complexes (Blundell *et al.*, 2002 ▸) is rather time-consuming without an automatic process and this is where cluster analysis plays a critical role (Bruno *et al.*, 2014 ▸). The combination of XRPD methods and multivariate analysis, such as principal-component analysis, provides a rapid and effective tool for studying the influence of ligands and pH on the crystallization process (Norrman *et al.*, 2006 ▸).

**Figure 4:**
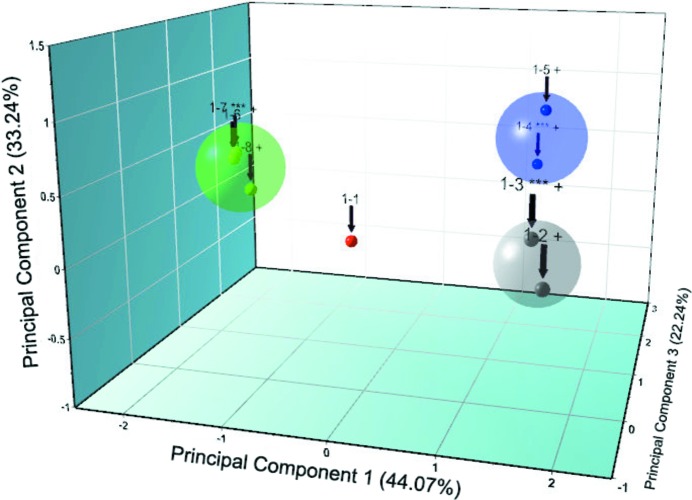
Cluster analysis of XRPD data for HI cocrystallized with 4-ethylresorcinol. Four distinct clusters were observed. The red cluster contains all data sets belonging to the new monoclinic symmetry *P*2_1(γ)_, the grey cluster contains all data sets belonging to the *P*2_1(α)_ symmetry, the blue cluster contains all data sets belonging to the monoclinic symmetry *C*2 and the green cluster contains all data sets belonging to the *P*2_1(β)_ symmetry. The numbers above each element correspond to the numbers of the samples. Data were collected on ID31 at ESRF [λ = 1.29994 (1) Å, RT].

In order to investigate the crystalline properties of our protein samples throughout the pH range of interest, cluster analysis was employed for the high-resolution synchrotron data sets as well as the laboratory data sets. This industrial analytical approach was performed using *HighScore Plus* (Degen *et al.*, 2014 ▸). Hierarchical cluster analysis produced four different groups, each corresponding to one of the different crystalline phases observed in our experiments, and also indicated the most representative samples for each cluster (marked with ‘***’ in Fig. 4 ▸). The cluster analysis for the samples from crystallization Series 1 is presented in Fig. 4 ▸.

## 3. Results   

The four distinct monoclinic polymorphs with the R_6_ molecular conformation identified from XRPD are now considered.

### 3.1. New monoclinic (*P*2_1_) polymorph γ   

**Figure 5:**
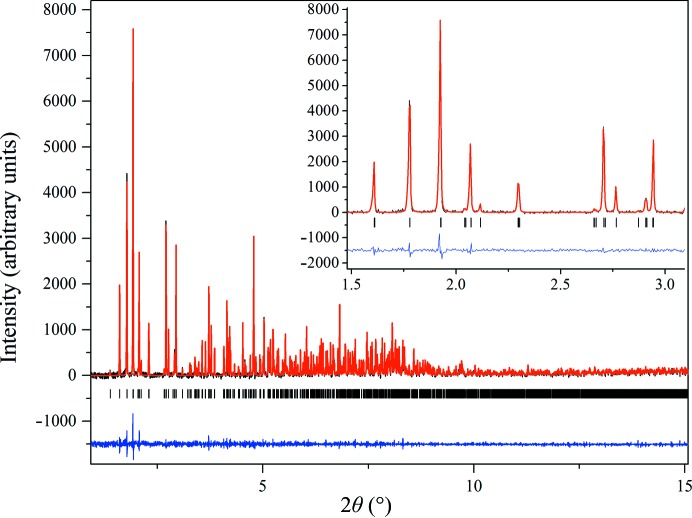
Pawley fit of HI cocrystallized with the ligand 4-ethylresorcinol [pH 5.10, polymorph *P*2_1(γ)_]. The data were collected at RT and a wavelength of 1.29994 (1) Å (ID31, ESRF). The black, red and lower blue lines represent the experimental data, the calculated pattern and the difference between the experimental and calculated profiles, respectively. The vertical bars correspond to Bragg reflections compatible with space group *P*2_1_.

In the case where HI was crystallized in the pH range 5.00–5.64 (Series 1) or 4.95–5.57 (Series 2), a new polymorph with monoclinic symmetry [referred to as* P*2_1(γ)_, unit-cell parameters *a* = 87.1323 (8), *b* = 70.294 (2), *c* = 48.064 (2) Å, β = 106.1729 (8)°] was observed. The same crystalline phase had been previously identified in our laboratory when insulin was crystallized with *meta*-cresol (*m*-cresol) or 4-nitrophenol (Valmas *et al.*, 2015 ▸). The Pawley fit to the data was satisfactory, with agreement factors of χ^2^ = 1.0324 and *R*_wp_ = 12.427% (Fig. 5 ▸). The data acquired for the *P*2_1(γ)_ polycrystalline samples extended to a resolution of ∼6.5 Å.

**Figure 6:**
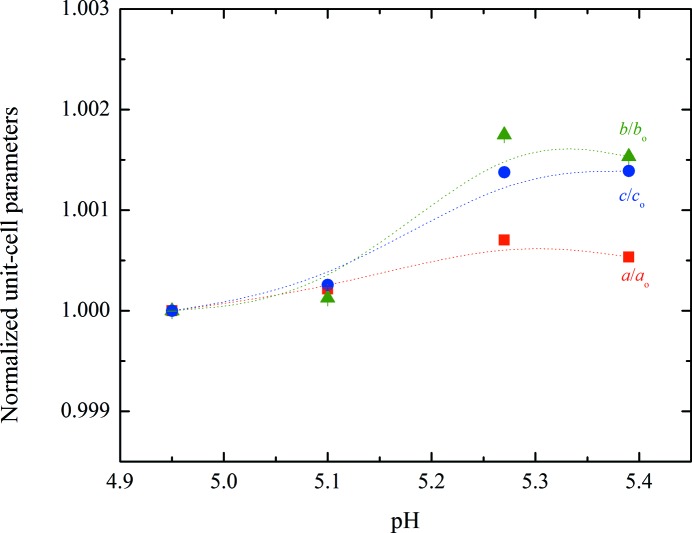
Evolution of the normalized unit-cell parameters of HI cocrystallized with 4-ethylresorcinol with increasing pH for the *P*2_1(γ)_ polymorph. The data employed were collected from Series 2 of crystalline samples on ID31 at ESRF [λ = 1.29994 (1) Å, RT].

**Figure 7:**
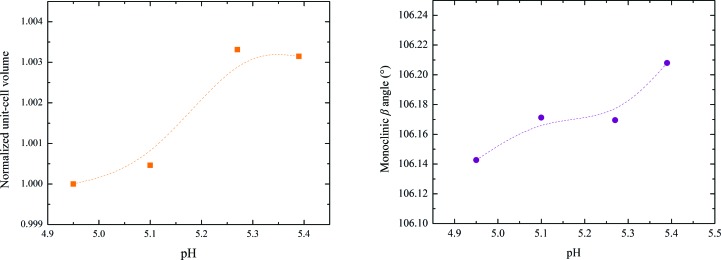
Evolution of the normalized unit-cell volume (left) and β angle (right) of HI cocrystallized with 4-ethylresorcinol with increasing pH for the *P*2_1(γ)_ polymorph. The data employed were collected from Series 2 of crystalline samples on ID31 at ESRF [λ = 1.29994 (1) Å, RT].

According to Matthews coefficient calculations (Matthews, 1968 ▸; Kantardjieff & Rupp, 2003 ▸), this phase contains 12 molecules (two hexamers) per asymmetric unit and 24 molecules (four hexamers) per unit cell, corresponding to a calculated solvent content of ∼39.3% (Matthews coefficient of 2.03 Å^3^ Da^−1^). The evolution of the normalized unit-cell parameters for Series 2 of samples measured at synchrotron sources is shown in Fig. 6 ▸, while the evolution of the unit-cell volume and β monoclinic angle are shown in Fig. 7 ▸. The complete structural model of the new *P*2_1(γ)_ polymorph has been determined by combining traditional single-crystal and emerging analytical XRPD methods and will be presented in a forthcoming publication (Karavassili *et al.*, 2015 ▸).

### 3.2. New monoclinic (*P*2_1_) polymorph α   

**Figure 8:**
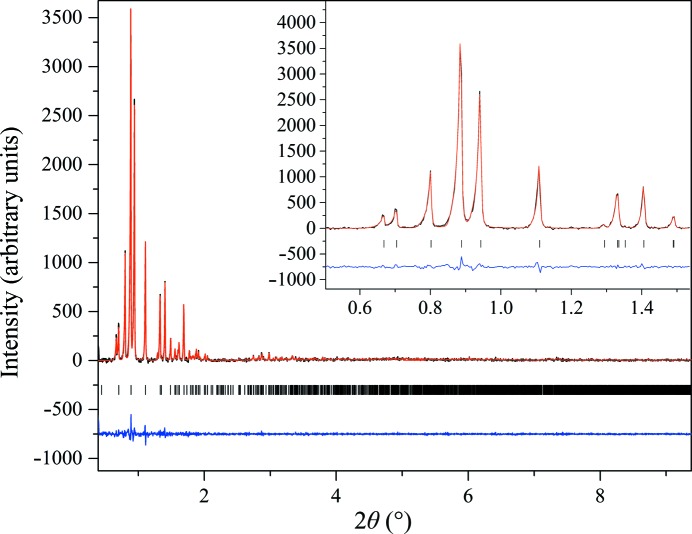
Pawley fit of HI cocrystallized with the ligand 4-ethylresorcinol [pH 5.80, *P*2_1(α)_ polymorph]. The data were collected at RT and a wavelength of 1.29994 (3) Å (ID31, ESRF). The black, red and lower blue lines represent the experimental data, the calculated pattern and the difference between the experimental and calculated profiles, respectively. The vertical bars correspond to Bragg reflections compatible with space group *P*2_1_.

Two HI samples crystallized at pH 5.64 and 5.80 adopted a previously unknown monoclinic phase [referred to as *P*2_1(α)_, unit-cell parameters *a* = 114.130 (7), *b* = 336.086 (3), *c* = 48.987 (5) Å, β = 101.935 (8)°]. This polymorph had previously been identified by our team in the case where HI was crystallized in the presence of phenol or resorcinol, but its complete structural model remained unresolved (Karavassili *et al.*, 2012 ▸). In this particular crystalline phase the *a* and *b* axes are considerably larger in comparison to the *c* axis; thus, all of the low-angle reflections used for indexing are of the form (*hk*0), so there is insufficient information to index a three-dimensional lattice (dominant-zone effect). Furthermore, the *b*:*a* ratio, which is very close to 3, complicates the indexing process even more since reflections such as (200) and (060) are very close in terms of 2θ and may not be observed as a result of peak overlap. In order to overcome this challenge, combined high-resolution data from the ID31 instrument and area-detector data collected at ID11 were used, as described in our previous study (Karavassili *et al.*, 2012 ▸). The Pawley fit to the data was satisfactory, with agreement factors of χ^2^ = 2.5038 and *R*_wp_ = 7.095% (Fig. 8 ▸). However, the data only extended to a resolution of ∼12 Å, which does not allow structure solution and refinement.

**Figure 9:**
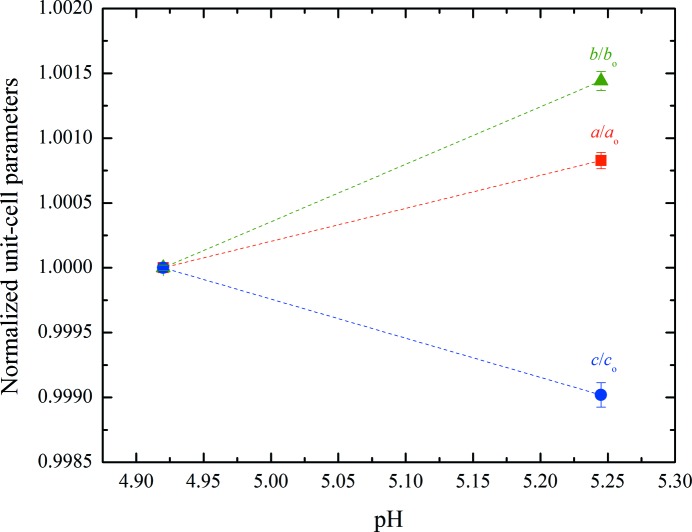
Evolution of normalized unit-cell parameters of HI cocrystallized with 4-­ethylresorcinol with increasing pH for the *P*2_1(α)_ polymorph. The data employed were collected from Series 1 of crystalline samples on ID31 at ESRF [λ = 1.29994 (3) Å, RT].

The unit-cell volume difference in the transition from *P*2_1(γ)_ to *P*2_1(α)_ corresponds to an increase of about 6.5-fold. This is a large unit-cell modification. This crystalline phase corresponds to one of the largest HI polymorphs that we have identified *via* XRPD methods to date, with the other one belonging to the *C*222_1_ symmetry {*V*(*C*222_1_) = 3 054 394 (63) Å^3^, *V*[*P*2_1(α)_] = 1 836 620 (73) Å^3^; Norrman & Schluckebier, 2007 ▸; Karavassili *et al.*, 2012 ▸}. The evolution of the normalized unit-cell parameters for the *P*2_1(α)_ samples is shown in Fig. 9 ▸.

### 3.3. Monoclinic (*C*2) polymorph   

**Figure 10:**
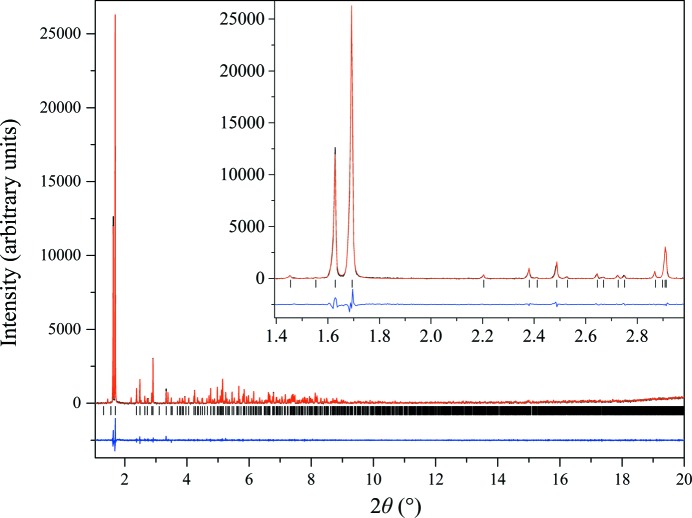
Pawley fit of HI cocrystallized with the ligand 4-ethylresorcinol (pH 6.23, *C*2 polymorph). The data were collected at RT and a wavelength of 1.29994 (3) Å (ID31, ESRF). The black, red and lower blue lines represent the experimental data, the calculated pattern and the difference between the experimental and calculated profiles, respectively. The vertical bars correspond to Bragg reflections compatible with space group *C*2.

HI crystallized in the pH range 5.97–6.23 (Series 1) or 5.88–6.21 (Series 2) adopted monoclinic symmetry [space group *C*2, unit-cell parameters *a* = 103.082 (7), *b* = 61.6636 (2), *c* = 63.5006 (4) Å, β = 117.417 (5)°]. The structure of this polymorph was first identified *via* XRPD methods (Norrman *et al.*, 2006 ▸) and was subsequently fully characterized *via* single-crystal methods (Norrman & Schluckebier, 2007 ▸; PDB entry 2olz). The Pawley fit to the data was very good, with χ^2^ = 1.0619 and *R*_wp_ = 4.728% (Fig. 10 ▸). The transition from *P*2_1(α)_ to *C*2 (Series 1) resulted in a vast reduction in the unit-cell volume, which was calculated to be about five times smaller, while the transition from *P*2_1(γ)_ to *C*2 (Series 2) was calculated as Δ*V*[*P*2_1(γ)_→*C*2]/Δ*V*[*P*2_1(γ)_] = +26.9%.

**Figure 11:**
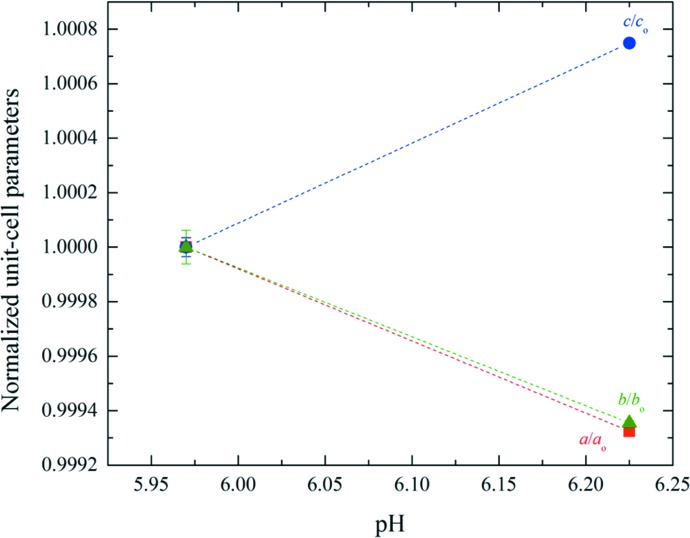
Evolution of normalized unit-cell parameters of HI cocrystallized with 4-­ethylresorcinol with increasing pH for the *C*2 polymorph. The data employed were collected from Series 1 of crystalline samples on ID31 at ESRF [λ = 1.29994 (3) Å, RT]

According to Matthews coefficient calculations (Matthews coefficient of 2.57 Å^3^ Da^−1^) this phase contains six molecules (one hexamer) per asymmetric unit and 24 molecules (four hexamers) per unit cell, while the solvent content is ∼52.11%. The evolution of the normalized unit-cell parameters for Series 1 of crystalline samples is shown in Fig. 11 ▸. The data acquired for the *C*2 polycrystalline samples extended to a resolution of ∼7 Å.

### 3.4. Monoclinic (*P*2_1_) polymorph β   

**Figure 12:**
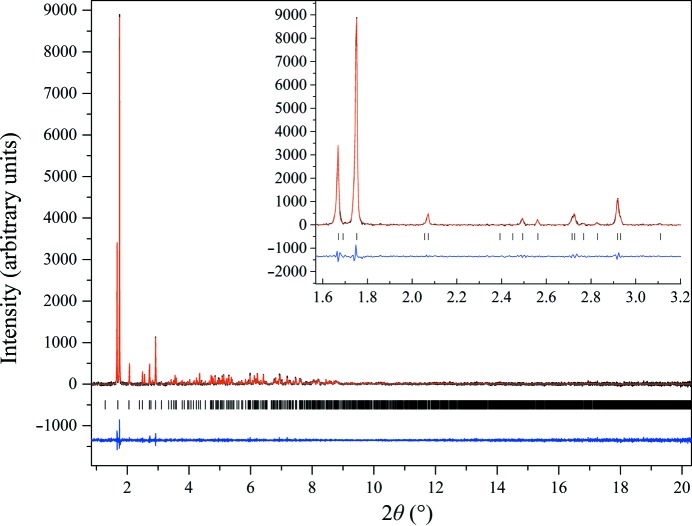
Pawley fit of HI cocrystallized with the ligand 4-ethylresorcinol [pH 7.19, monoclinic crystal system, *P*2_1(β)_ polymorph]. The data were collected at RT and a wavelength of 1.29994 (3) Å (ID31, ESRF). The black, red and lower blue lines represent the experimental data, the calculated pattern and the difference between the experimental and calculated profiles, respectively. The vertical bars correspond to Bragg reflections compatible with space group *P*2_1_.

**Figure 13:**
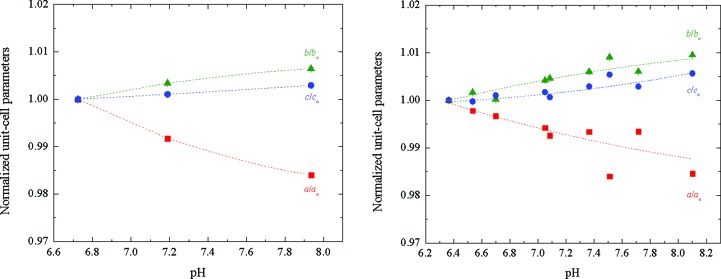
Evolution of normalized unit-cell parameters of HI cocrystallized with 4-ethylresorcinol with increasing pH for the *P*2_1(β)_ polymorph. The data employed were collected from Series 1 (left) and Series 2 (right) of samples on ID31 at ESRF [λ = 1.29994 (3) Å, RT and λ = 1.29994 (1) Å, RT, respectively].

In the pH range 6.73–7.94 (Series 1) or 6.70–8.10 (Series 2) the crystals obtained belonged to the monoclinic symmetry *P*2_1_ [referred to as *P*2_1(β)_, unit-cell parameters *a* = 62.8231 (7), *b* = 62.1078 (5), *c* = 47.8362 (6) Å, β = 111.6913 (9)°], a previously characterized polymorph (Derewenda *et al.*, 1989 ▸; Smith *et al.*, 2000 ▸; PDB entry 1evr). The Pawley fit to the data was satisfactory, with χ^2^ = 1.8545 and *R*_wp_ = 7.926% (Fig. 12 ▸). The difference in the unit-cell volume during the transition from *C*2 to *P*2_1(β)_ corresponds to a reduction by a factor of ∼2. This polymorph is characterized by a smaller crystallographic unit-cell volume in comparison to the rest of the identified polymorphs. According to Matthews coefficient calculations, this polymorph contains six molecules (one hexamer) per asymmetric unit and 12 molecules (two hexamers) per unit cell, while the solvent content is ∼50.32% (Matthews coefficient of 2.48 Å^3^ Da^−1^). The evolution of the normalized unit-cell parameters for the two series of samples measured are shown in Fig. 13 ▸. The data acquired for the *C*2 polycrystalline samples extended to a resolution of ∼6 Å.

## 4. Discussion   

**Figure 14:**
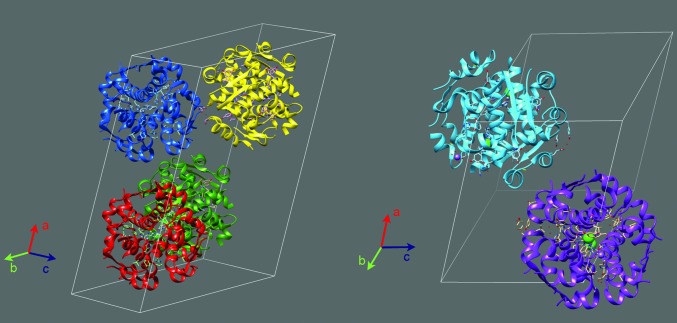
Models of the unit-cell contents for the known *C*2 (PDB entry 2olz, left) and *P*2_1(β)_ (PDB entry 1evr, right) polymorphs. The white lines correspond to the axes of the unit cells. Different colours correspond to distinct HI hexamers. Chloride ions are coloured green [*P*2_1(β)_]. The models were created with *USCF Chimera*.

In this work, we present a systematic crystallographic study of HI cocrystallized with the organic ligand 4-ethylresorcinol within the pH range 4.5–8.2. Crystallization experiments were reproduced twice and resulted in polycrystalline precipitates, which were employed for XRPD measurements. Data analysis resulted in an accurate mapping of the symmetry and unit-cell parameters for all observed distinct monoclinic crystalline phases. Four different polymorphs were identified, which belonged to two different space groups (*P*2_1_ and *C*2). Two of these polymorphs [*C*2 and *P*2_1(β)_] were structurally known (Fig. 14 ▸) and two were first reported by our team in previous studies [*P*2_1(α)_ and *P*2_1(γ)_]. To date, only their unit-cell parameters and space groups have been reported (Karavassili *et al.*, 2012 ▸; Valmas *et al.*, 2015 ▸); complete structural models are not yet available in the PDB.

The monoclinic polymorphs observed in this study belong to the R_6_ molecular conformation (Norrman & Schluckebier, 2007 ▸; Smith *et al.*, 2000 ▸). Insulin ligand binding clearly exhibits an allosteric behaviour (Bentley *et al.*, 1976 ▸; Ciszak & Smith, 1994 ▸; Smith *et al.*, 1996 ▸; Whittingham *et al.*, 1995 ▸; Derewenda *et al.*, 1989 ▸; Smith & Dodson, 1992 ▸). The transitions between the extended (T) and α-helical (R) conformations involve three states. *In vivo*, the insulin hexamer exists in the T_6_ state, whereas the addition of anions (for example chloride or thiocyanate) and phenolic ligands induces the T_3_R_3_ and R_6_ states (Dunn, 2005 ▸; Huus *et al.*, 2006 ▸). The T-to-R transitions require the transformation of residues B1–B8 from an extended to a helical conformation (Smith *et al.*, 1984 ▸; Derewenda *et al.*, 1989 ▸; Ferrari *et al.*, 2001 ▸). This transformation creates hydrophobic pockets in which phenol and its derivatives bind (Huus *et al.*, 2006 ▸). There are three hydrophobic pockets in the T_3_R_3_ hexamer and six in the R_6_ hexamer (Dunn, 2005 ▸). The binding interactions of ligands in the phenolic pockets result in the T_3_R_3_ and R_6_ conformations, which are further stabilized by the binding of certain anions that do not stabilize the T_6_ state, such as halides, pseudo-halides and organic carboxylates (Bentley *et al.*, 1976 ▸; Dunn, 2005 ▸; Rahuel-Clermont *et al.*, 1997 ▸; Huus *et al.*, 2006 ▸). Therefore, as most pharmaceutical preparations contain phenolic derivatives as preservatives, the HI molecules have the T_3_R_3_ and R_6_ conformations (Rahuel-Clermont *et al.*, 1997 ▸; Ferrari *et al.*, 2001 ▸). As the stability level decreases from R to T, with R_6_ being the most stable conformation (Rahuel-Clermont *et al.*, 1997 ▸), the existence of these conformations may function *in vivo* to create a balance between the stable storage and the gradual release of the active monomer. Moreover, the allosteric transition at the level of the monomer could be important in the binding affinity between insulin and its receptor (Bloom *et al.*, 1995 ▸).

With regard to drug development, the crystal size needs to be much smaller than 50 µm in order for pharmaceutical formulations to be easily injectable and to limit possible immunogenicity reactions (Basu *et al.*, 2004 ▸). Control of the crystal size can be achieved by different methods such as variation of the concentration of the crystallizing agents (Collings *et al.*, 2010 ▸) or polymeric coating, which inhibits crystal growth and reduces the particle size (Rabinow, 2004 ▸). Finally, pharmaceutical formulations need to contain iso­granular and isometric crystals, which means that there should be homogeneity in crystal size (Collings *et al.*, 2010 ▸). After years of exponential development in terms of instrumentation and experiment design, XRPD is now efficient at the rapid and accurate characterization of numerous protein microcrystalline precipitates of such size with regard to homogeneity and purity, whereas the extraction of accurate unit-cell parameters, as reported by our team in the present and previous studies, can indicate minor or major structural modifications.

In the case of injected treatments, it would be desirable to develop more effective formulations. This could be achieved by reducing the crystal dissolution rate and increasing the amount of active ingredient per dose. pH variation can result in distinct polymorphs with different physicochemical properties such as density, solubility and stability (Rabinow, 2004 ▸). These characteristics can further affect their dissolution rate and thus their bioavailability. Ultralente (Eli Lilly), which has been one of the most essential insulin compounds, is a suspension of insulin microcrystals that dissolve slowly following subcutaneous injection (Wagner *et al.*, 2009 ▸). Therefore, the identification of new polymorphs could lead to the optimization of existing formulations or the design of advanced ones with a different action depending on the needs of patients. It could also lead to the creation of new forms that are associated with alternative methods of administration, such as formulations with sustained release or formulations for inhaled administration (Basu *et al.*, 2004 ▸).

**Figure 15:**
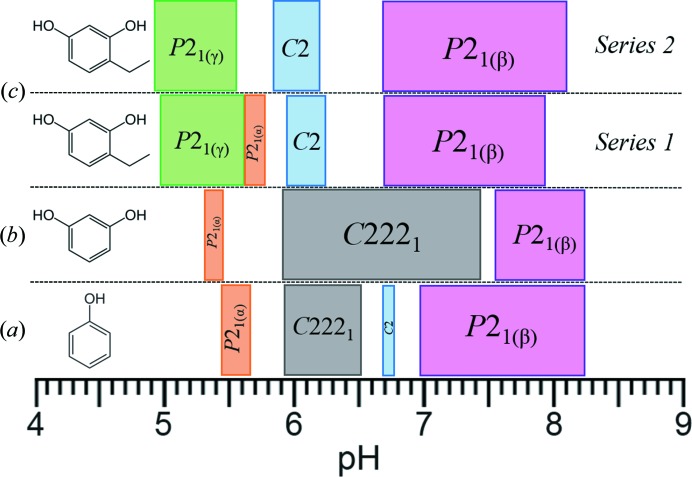
Phase diagram of HI cocrystallized with ligands: (*a*) phenol, (*b*) resorcinol (Karavassili *et al.*, 2012 ▸) and (*c*) the two successive series of experiments with 4-ethylresorcinol, indicating the reproducibility of the results reported here. The figure illustrates how the phase diagram of HI varies with the distinct ligands involved in cocrystallization. The selected ligands are shown on the left.

In this study, we report the identification of two new HI crystalline phases, *P*2_1(γ)_ and *P*2_1(α)_, at low pH levels (∼4.8–5.8). The *P*2_1(α)_ polymorph has previously been identified by our team in the presence of the ligands phenol and resorcinol in approximately the same pH range (Karavassili *et al.*, 2012 ▸). This phase was observed only in one of the two crystallization series (Series 1, as illustrated in Fig. 15 ▸). This discrepancy may be related to seeding and the kinetics of phase formation. Moreover, the two sample series are characterized by different time intervals between crystallization and synchrotron data collection. We note that related effects have been observed in the past for other HI complexes. In addition, the *P*2_1(γ)_ polymorph has been identified in the cases of four different ligands, two of them recently presented (Valmas *et al.*, 2015 ▸). The specific ligands are 4-bromoresorcinol, 4-chororesorcinol, *m*-cresol and 4-nitrophenol. These two polymorphs appear in an acidic environment, while the previously known polymorph *P*2_1(β)_ appears in the pH range ∼6.8–8.1 (Fig. 15 ▸).

**Figure 16:**
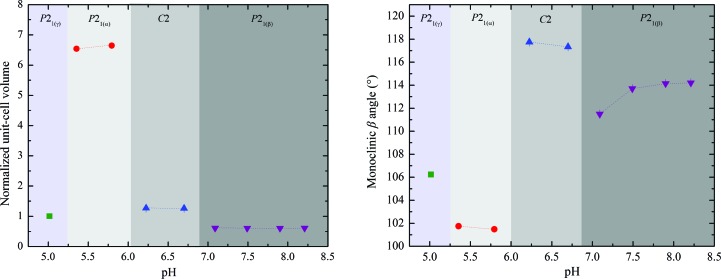
Evolution of the normalized unit-cell volume (left) and monoclinic β angle (right) of HI cocrystallized with 4-ethylresorcinol with increasing pH.

The results described in the present article illustrate the extent of polymorphism of HI. The change in the volume and β angle over the pH range is shown in Fig. 16 ▸. It is obvious that the crystalline phase *P*2_1(α)_ has a much larger unit-cell volume owing to the long *b* axis.

The new *P*2_1(γ)_ polymorph has a unit-cell volume that is larger by 38% in comparison to the known *P*2_1(β)_ polymorph. The extended area is occupied by two additional hexamers [a total of four hexamers in the unit cell and 39.3% solvent content, while in the *P*2_1(β)_ polymorph there are two hexamers per unit cell and 50.32% solvent content]. Thus, the polymorph is characterized by a more dense packing of the insulin hexamers with much stronger intermolecular contacts. This characteristic is important for the future production of microcrystalline insulin drugs because it confers the advantage of a higher mass per volume loading, which is crucial when higher dosing is required (Rabinow, 2004 ▸). This polymorph could provide the possibility of supplying a larger amount of insulin with smaller drug doses and thereby reducing the frequency of dosing in people with diabetes.

Our results demonstrate that systematic screening of crystallization conditions in combination with synchrotron and laboratory XRPD yields an exact and unambiguous picture of the crystallization behaviour of insulin. Even around its pI (∼5.9), where its solubility is lowest and the growth of macroscopic crystals suitable for single-crystal X-ray structure determination is less likely to succeed, we observed sufficient numbers of crystals, although these were smaller than those obtained at lower and higher pH values (Fig. 1 ▸).

Further crystallization experiments are currently in progress in order to improve the resolution of the collected XRPD profiles. We aim to obtain single crystals in order to solve and refine the structures of the new as well as the known polymorphs. This is necessary in order to identify the protein structure in each polymorph as well as the ligand-binding sites. We believe that this kind of systematic approach further extends the applicability of XRPD methods for macromolecular crystal screening in a wide range of crystallization conditions.

## Figures and Tables

**Table 1:** Description of the data-collection parameters for the XRPD experiments

Space group	X-ray source	Beamline	Detector	Resolution ()	Capillary diameter (mm)	No. of exposures per position	Exposure time per scan	No. of scans
*P*2_1()_	Synchrotron radiation	ID31, ESRF	APD†	6.5	1.0	2	2min	6
		MS-X04SA, SLS	Mythen‡	6.5	0.5	35	2s	12
*P*2_1()_	Synchrotron radiation	ID31, ESRF	APD	12	1.0	2	2min	6
*C*2	Synchrotron radiation	ID31, ESRF	APD	7	1.0	2	2min	6
		MS-X04SA, SLS	Mythen	9	0.5	35	2s	12
*P*2_1()_	Synchrotron radiation	ID31, ESRF	APD	6	1.0	2	2min	6
		MS-X04SA, SLS	Mythen	9	0.5	35	2s	12

† Nine Si(111) analyser crystals each followed by a point detector [avalanche photodiode (APD) detectors].

‡ Modular (each module covering 5 in 2) one-dimensional solid-state Si microstrip detector (Mythen II) covering 120 in 2.
